# Hippo in smooth muscle - a therapeutic target in vascular diseases driven by aging and hypertension

**DOI:** 10.3389/fphys.2025.1674714

**Published:** 2025-10-07

**Authors:** Sebastian Albinsson, Catarina Rippe, Fatima Daoud, Joakim Armstrong Bastrup, Johan Holmberg, Thomas A. Jepps, Karl Swärd

**Affiliations:** ^1^ The Cellular Biomechanics and Molecular Vascular Physiology Groups, Department of Experimental Medical Science, Lund University, Lund, Sweden; ^2^ Department of Physiology and Biochemistry, University of Jordan, Amman, Jordan; ^3^ Vascular Biology Group, Department of Biomedical Sciences, University of Copenhagen, Copenhagen, Denmark

**Keywords:** hippo, YAP, TAZ, myocardin, smooth muscle, aneurysm, atherosclerosis, Lats2

## Abstract

**Introduction:**

The Hippo signaling pathway is a key regulator of cellular growth and organ size, acting through the transcriptional coactivators YAP and TAZ. These proteins shuttle between the nucleus and cytoplasm in response to Hippo pathway activity, which, when active, leads to cytoplasmic sequestration and degradation of YAP/TAZ, preventing them from initiating gene transcription. Although initially studied in development and cancer, recent research has revealed crucial functions for YAP and TAZ in the adult vascular wall.

**Scope of the review:**

This review discusses emerging insights into the roles of Hippo signaling and its downstream effectors YAP and TAZ in adult vascular smooth muscle cells (SMCs) and endothelial cells (ECs), with an emphasis on their physiological and pathological relevance.

**Key findings:**

In SMCs, YAP and TAZ are vital for maintaining contractile identity by regulating expression of SMC contractile proteins. Inducible deletion of YAP/TAZ in adult SMCs results in impaired contractility, hypotension, and spontaneous arterial aneurysms. Despite these findings, the role of upstream Hippo signaling in SMCs remains poorly understood, and its therapeutic potential is underexplored. In ECs, YAP and TAZ respond to disturbed flow patterns by promoting a pro-atherogenic gene expression profile, contributing to increased atherosclerotic burden in hypercholesterolemic conditions.

**Discussion and conclusion:**

Targeting Hippo-YAP/TAZ signaling in vascular cells represents a promising yet complex strategy for treating vascular diseases. The key challenge lies in achieving precise, cell-specific, and temporally controlled modulation that enhances beneficial effects, such as aneurysm protection and arterial repair, while minimizing off-target or adverse effects in non-vascular tissues.

## 1 Introduction

### 1.1 The Hippo signaling pathway and YAP and TAZ

**FIGURE 1:**
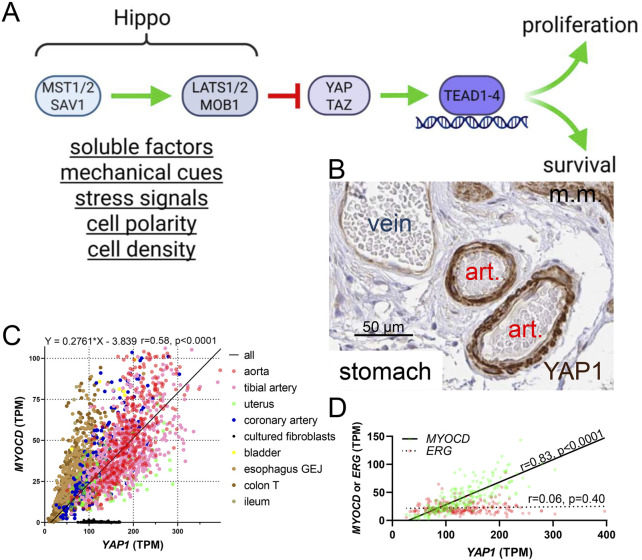
The Hippo pathway and YAP1 expression in smooth muscle cells. **(A)** shows an overview of the Hippo pathway upstream of the transcriptional coactivators YAP and TAZ. When Hippo activity is high, YAP and TAZ activity is low, and *vice versa*. Inside nuclei, DNA-binding partners of YAP and TAZ are the TEADs (TEAD1-4). Broad categories of regulators of Hippo are underlined below the MST and LATS complexes. **(B)** shows staining for YAP1 in small submucosal arteries in the stomach (from the Human Protein Atlas, HPA). Art.: artery, m.m.: muscularis mucosa. The scale bar in this, and the remainder of the immunohistochemistry images, represents 50 µm. **(C)** shows that *YAP1* correlates with myocardin (*MYOCD*) across human tissues (GTEx RNA-seq data, n ≥ 132). Significant (P < 0.0001) correlations are present throughout, with the singular exception of cultured fibroblasts (the key to the symbols is shown to the right of the graph) which were included for comparison. Lack of association in culture may be due to lack of a three-dimensional *in vivo* environment with natural cell-cell and cell-matrix interactions. The equation for the regression line is given above (all tissues included in analysis), as are the Spearman r and p values. TPM: transcripts per million. **(D)** shows a comparison of the *YAP1* vs *MYOCD* (green symbols) and *YAP1* vs *ERG* (red symbols) correlations in human coronary artery (GTExPortal data, n = 240).

Hippo refers to the MST/SAV (official gene symbols: *STK3*, *4*, and *SAV1*) and LATS/MOB (*LATS1*, *LATS2*, and *MOB1A*/*B*) kinase complexes that act upstream of the transcriptional coactivators YAP (*YAP1*) and TAZ (*WWTR1*) to inhibit their activity *via* phosphorylation (Figure 1A). Phosphorylation of YAP and TAZ leads to their cytoplasmic retention and degradation. When YAP and TAZ are dephosphorylated (Hippo activity is low) they enter the nucleus where they bind to one of four TEAD transcription factors to drive gene expression. Gene targets are context-dependent, but those important for cell proliferation and survival are featured consistently. The pathway is named after the Hippo (*hpo*) gene in *Drosophila* which corresponds to the MST kinases in mammals (Harvey and Tapon, 2007). When *Drosophila hpo* is inactivated, flies develop abnormally large organs due to excessive cell proliferation and reduced apoptosis, and hence the name Hippo. Given its role in growth control, Hippo has received considerable attention, and the LATS kinases have been dubbed tumor suppressors (Large Tumor Suppressor Kinase 1 and 2). The focus of the present review is on the function of Hippo-YAP/TAZ in the vascular wall, and a key aim is to discuss how realistic it is to inhibit Hippo for anti-aneurysmal therapy.

The number of inputs into the Hippo pathway is staggering, and the full range of known regulatory mechanisms are comprehensively covered in recent reviews (Driskill and Pan, 2023; Fu et al., 2022; Guo et al., 2025; Kobayashi et al., 2023). Soluble factors, stress signals, cell polarity and density, as well as mechanical cues represent broad categories of regulators (Figure 1A) (Fu et al., 2022). A key feature of YAP and TAZ, likely to be particularly important in the vascular wall, is their exquisite sensitivity to mechanical signals. Because SMCs are arranged helically around the arterial lumen, they are stretched when blood pressure increases, and arteries are exposed to both steady and pulsative pressure generated by the heart throughout life. Given that YAP and TAZ are stabilized upon activation, through evasion of proteasomal degradation, blood pressure is predicted to contribute to higher expression in arteries (where blood pressures range from 50 to 140 mmHg) than in veins (≈10–15 mmHg). This is indeed seen, with a particularly prominent difference of YAP staining in the vascular media where SMCs reside (Figure 1B).

A handful of studies published in 2011–2012 first identified mechanical cues as a crucial aspect of YAP and TAZ activation. Using micropatterned substrates and matrices with different rigidity, Dupont et al. (2011) demonstrated that stiff surfaces promoted nuclear entry and activation of YAP and TAZ. It was also found that depolymerization of actin (Latrunculin A) and inhibition of the monomeric GTPase Rho (C3 toxin) counteracted YAP/TAZ activation by matrix stiffness. It was argued that actomyosin-based contractility *per se* drives YAP/TAZ activation, because treatment with the myosin inhibitor blebbistatin turned YAP and TAZ off, like depolymerization of actin-filaments did. Most of these findings have since been confirmed and extended in other laboratories, but one point initially remained contentious, namely to what extent mechanical cues act independently of Hippo. While Dupont et al. argued that mechanics act in parallel with Hippo, others provided evidence that LATS activity is required (Wada et al., 2011; Sansores-Garcia et al., 2011; Zhao et al., 2012). More recent work on the role of Rap2 for inactivation of YAP/TAZ on soft matrices strengthened support for Hippo involvement (Meng et al., 2018), but also acknowledged cytoskeletal tension as an independent and direct input on YAP and TAZ. Importantly, YAP/TAZ activation by other mechanical modalities, including cyclic stretch (Codelia et al., 2014; Zhang et al., 2023; Yamashiro et al., 2020) and disturbed flow (Gegenfurtner et al., 2018) has been demonstrated. Our own work showed that the transcriptomic response to chronic isotonic vascular stretch depends on YAP and TAZ (Daoud et al., 2022a).

Actin dynamics, Rho family GTPases, and Rho-associated kinases are critical effector molecules for control of vasomotor tone (Sward et al., 2003; Somlyo and Somlyo, 2003; Loirand et al., 2006) and for YAP/TAZ activation (Dupont et al., 2011; Wada et al., 2011; Sansores-Garcia et al., 2011; Zhao et al., 2012; Meng et al., 2018). Moreover, considering that elevated blood pressure is the risk factor that makes the largest contribution to the global burden of disease, and given that YAP and TAZ play a role in the transcriptomic response to pressure (Codelia et al., 2014; Zhang et al., 2023; Daoud et al., 2022a), aims in our laboratories include to elucidate artery-specific aspects of YAP/TAZ regulation and to test if the Hippo pathway is targetable for therapy in arterial disease.

## 2 Summary of known functions of Hippo-YAP/TAZ in vascular cells

### 2.1 YAP and TAZ in SMCs

Interest in Hippo and YAP/TAZ in our groups originated with the observation (Albinsson and Swärd circa 2017) that *YAP1* correlates with the transcriptional coactivator myocardin (*MYOCD*), a master regulator of SMC differentiation (Miano, 2015; Sward et al., 2016), across human tissues. We used RNA-seq data from GTExPortal (GTEx Consortium et al., 2013; GTEx Consortium, 2015) with the aim of identifying novel target genes of MYOCD (Krawczyk et al., 2015; Liu L. et al., 2021; Rippe et al., 2021; Sward et al., 2019; Zhu B. et al., 2018), leading to the discovery of this robust association (Figure 1C). No correlation was observed for the endothelial cell (EC) transcription factor *ERG* as shown for the coronary artery (Figure 1D). Moreover, immunohistochemistry in the Human Protein Atlas (Uhlen et al., 2015) demonstrated staining for YAP in the arterial media (Figure 1B, brown) with less staining in endothelial cells and veins. Considering the fundamental importance of MYOCD and its nuclear binding partner serum response factor (SRF) for SMC differentiation (Miano, 2015; Sward et al., 2016), our initial observations led us to create inducible and SMC-specific knockouts of YAP and TAZ, uncovering a range of severe phenotypes involving gastrointestinal (Daoud et al., 2021) and urogenital (Liu L. et al., 2023) SMC organs as well as arteries (Daoud et al., 2022a; Arevalo Martinez et al., 2023). These studies are described below. Our foundational finding that *YAP1* and *MYOCD* correlate across human tissues is probably explained, at least in part, by YAP/TEAD-dependent control of *MYOCD* expression (Arevalo Martinez et al., 2023; Creemers et al., 2006; Wen et al., 2019) *via* a distal enhancer (Creemers et al., 2006), but we do not rule out *YAP1* enrichment in SMCs relative to other vascular cells by some other unknown mechanism.

*In vivo* studies using SMC-specific knockouts of Hippo pathway mediators, including MST and LATS kinases, are scarce, and further work using inducible, lineage-specific Cre lines is necessary to clarify contributions to vascular homeostasis, and disease. While the role of Hippo in SMCs of systemic arteries represents an important knowledge gap, YAP and TAZ, the effectors of Hippo, do play critical roles as demonstrated using cell type-specific knockout paradigms. We therefore start here by summarizing the phenotypes of mice with inducible SMC-specific inactivation of YAP and TAZ and then speculate on the roles that Hippo might play. An important point to first consider is that YAP and TAZ are very similar in many regards, and that they can substitute for each other. Our experience, and that of others (Wang et al., 2020), is that isolated knockout of either YAP or TAZ in adult mice has rather limited effects compared to dual knockout, supporting coactivator redundancy.

Conditional deletion of YAP/TAZ in smooth muscle reveals essential roles for these transcriptional coactivators during development and for adult tissue function. Constitutive deletion of YAP, without concurrent deletion of TAZ, in SMCs and cardiomyocytes using the *SM22α-Cre* driver causes perinatal lethality, reflecting its requirement in cardiovascular development (Wang Y. et al., 2014). Embryos with YAP-deficient smooth muscle develop hypoplastic arteries with reduced medial thickness and impaired proliferation of SMCs. This points to a fundamental role for YAP in driving smooth muscle development and vessel wall formation during embryogenesis. However, contractile differentiation of SMCs was largely maintained in this model suggesting that YAP, in the presence of TAZ, is dispensable for the establishment of the contractile gene program during development but essential for proliferative expansion and structural maturation of the vessel wall.

**FIGURE 2:**
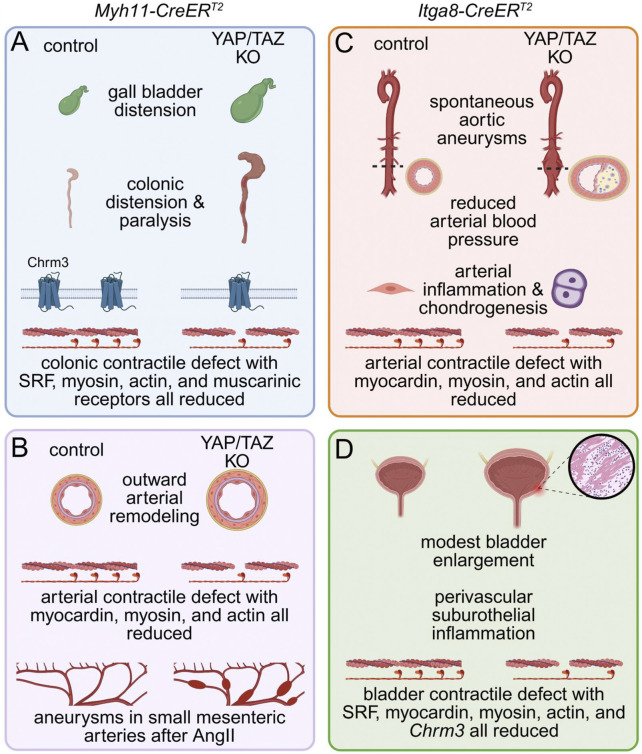
Phenotypes of mice with inducible and SMC-specific inactivation of YAP and TAZ. SMC-specific knockout of YAP and TAZ was achieved using either the *Myh11-CreER* ^*T2*^ **(A,B)** or the *Itga8-CreER* ^*T2*^ **(C,D)** strain and the floxed *Yap1* and *Wwtr1* alleles. While the former model causes recombination in all SMCs, including those in the gastrointestinal (GI) tract, the latter spares GI SMCs and favors deletion in arterial and other SMCs. When *Myh11-CreER* ^*T2*^ was used for YAP and TAZ knockout **(A,B)**, mice became kyphotic with reduced food consumption and defecation, forcing termination within 17 days after the first tamoxifen injection. Autopsy revealed colonic and gall bladder distension, and myography uncovered a severe contractile defect in the distal GI tract that was also supported by RNA-sequencing and proteomics **(A)**. Arteries from knockouts **(B)** showed outward remodeling and impaired contractility with reduced expression of myocardin and SMC differentiation markers. To circumvent a severe GI phenotype *Itga8-CreER* ^*T2*^ strain was instead used for YAP/TAZ knockout **(C,D)**. This resulted in spontaneous aortic aneurysm formation, characterized by aortic bulging, neointima formation, infiltration of inflammatory cells, and increased chondrogenesis, but reduced arterial blood pressure. Arterial force development was severely impaired and myocardin, myosin and actin were all reduced. In this model, the urinary bladder was modestly enlarged and featured perivascular suburothelial inflammation along with reduced detrusor muscle contractility **(D)**. SRF, myocardin and contractile markers were all reduced, mimicking bladder transcriptomic changes seen after *Myh11-CreER* ^*T2*^ knockout.

Considering the developmental phenotype and the mutual redundance of YAP and TAZ in adult smooth muscle, we used a novel mouse model with inducible, smooth muscle-specific deletion of both YAP and TAZ to determine their importance in adult mice (Daoud et al., 2021). This model was generated by crossing *Myh11-CreER* ^*T2*^ transgenic mice (Wirth et al., 2008) with mice carrying floxed alleles of *Yap1* and *Wwtr1* (Reginensi et al., 2013). The most rapid phenotype was observed in the gastrointestinal tract, where tamoxifen-induced knockout of YAP/TAZ in adult mice produced colonic pseudo-obstruction (Daoud et al., 2021). At 9–11 days after the first tamoxifen injection, KO mice presented with marked gall bladder distention, colonic dilation, and near-complete loss of gastrointestinal peristalsis (Figure 2A). Supporting translatability, gall bladder phenotypes are indeed tied to the *WWTR1* locus in man (https://pheweb.org/UKB-SAIGE/gene/WWTR1). Colonic contractile responses to muscarinic agonists were essentially abolished in knockouts, and transcriptional profiling revealed broad downregulation of M_2_ and M_3_ muscarinic receptors, contractile markers such as *Myh11* and *Acta2*, as well as transcriptional regulators like *Srf* (Figure 2A). Although the thickness of the intestinal smooth muscle layer was reduced in YAP/TAZ knockouts, the total smooth muscle area was maintained, suggesting distension rather than SMC loss at this early timepoint.

In the vascular system, inducible deletion of YAP and TAZ in smooth muscle using the *Myh11-CreER* ^*T2*^-driver triggers a profound transcriptional reprogramming, characterized by downregulation of contractile and myocardin-dependent genes, and upregulation of inflammatory, osteochondrogenic, and adipogenic pathways (Daoud et al., 2022a; Wang et al., 2020). As early as 9–11 days following tamoxifen induction, arteries from YAP/TAZ knockout mice exhibit markedly reduced vasoconstrictor responses, attenuated myogenic tone and mechanosensing in smooth muscle, along with increased vascular compliance (Daoud et al., 2022a) (Figure 2B). These changes, which occur independently of blood pressure, elevate wall stress by promoting vessel dilation and reducing medial thickness. The elevated mechanical burden is likely to promote vascular injury and contribute to the initiation and progression of vascular disease (Belhoul-Fakir et al., 2023). Consistent with these findings, hypertensive mice (AngII) with smooth muscle–specific deletion of YAP/TAZ developed rapidly progressing aneurysms in small mesenteric arteries within 11 days (Daoud et al., 2022a) (Figure 2B, bottom). These aneurysms exhibited elastin degradation, adventitial hyperplasia, and inflammatory cell infiltration, features that are characteristic of pathological vascular remodeling. Importantly, in this model with its limited time course, normotensive YAP/TAZ knockout mice did not develop aneurysms. This suggests that while deleting YAP/TAZ in smooth muscle is sufficient to drive aneurysm formation, the pathological changes require time to manifest unless mechanical stress is elevated. Thus, hypertension accelerates the process, revealing the inability of YAP/TAZ-deficient vessels to maintain structural integrity under increased load.

To further dissect the long-term vascular consequences of YAP/TAZ deficiency, while avoiding effects associated with Cre activity in visceral smooth muscle, we employed an alternative model using the *Itga8-CreER* ^*T2*^ driver (Arevalo Martinez et al., 2023). *Itga8-CreER* ^*T2*^ drives recombination more selectively in vascular SMCs, with minimal activity in gastrointestinal smooth muscle (Warthi et al., 2022). This spatial specificity allowed for focused investigation of vascular phenotypes without the confounding effects of colonic pseudo-obstruction prominent in *Myh11-CreER* ^*T2*^-driven knockouts.

Using this model, we observed that vascular SMC-specific deletion of YAP and TAZ led to the development of spontaneous abdominal aortic aneurysms within 2 weeks of tamoxifen induction (Figure 2C), despite the mice being hypotensive and normolipidemic (Arevalo Martinez et al., 2023). This distinguishes the model from most existing genetic mouse models of aneurysmal disease, which typically require additional stressors such as hypertension and/or hyperlipidemia (Daugherty et al., 2000). The vascular pathology involved loss of SMC contractile differentiation, inflammatory cell infiltration, and substantial proteoglycan accumulation in the aortic wall, all of which are characteristics of human aneurysmal disease (Arevalo Martinez et al., 2023). Blood pressure reduction likely reflects decreased peripheral vascular resistance from impaired arterial contractility, though other mechanisms may contribute. Unlike the *Itga8-CreER* ^*T2*^ model, which showed lower blood pressure 3 weeks after induction, the *Myh11-CreER* ^*T2*^ model showed no reduction 8–10 days post-induction. Data beyond 2 weeks are lacking due to poor viability. Associations between genetic variants in the Hippo pathway and blood pressure traits in the human population are discussed below (see 2.5.).

At the molecular level, transcriptomic and proteomic profiling revealed downregulation of *Myocd* and its downstream contractile targets (*Myh11*, *Acta2*) (Arevalo Martinez et al., 2023), indicating that YAP/TAZ deletion disrupts myocardin-SRF signaling, a central driver of smooth muscle identity (Wang et al., 2002; Du et al., 2003; Chen et al., 2002). Although diminished contractility is unlikely to fully explain aneurysm formation, it may contribute to vessel wall weakening and progressive dilation. Importantly, activation of proinflammatory signaling was evident in the aortic wall following YAP/TAZ deletion (Arevalo Martinez et al., 2023). This included upregulation of the cytosolic DNA sensor STING and its downstream effector kinase TBK1, both key mediators of innate immune responses (Arevalo Martinez et al., 2023; Sladitschek-Martens et al., 2022). Elevated expression of STING and phosphorylated TBK1 was accompanied by increased transcription of STING-dependent target genes and marked infiltration of both innate and adaptive immune cell populations, indicating a sustained inflammatory response (Arevalo Martinez et al., 2023). Notably, prior to the onset of inflammation or overt structural changes, there was a pronounced induction of the chondrogenic transcription factor SOX9 in medial smooth muscle cells, along with accumulation of aggrecan and other proteoglycans, occasionally causing outright chondrogenesis (Figure 2C). These extracellular matrix changes were temporally the earliest detectable abnormalities and suggest that SOX9-mediated matrix remodeling may represent a primary event in pathogenesis, potentially acting as a trigger for subsequent inflammatory activation and vascular degeneration. Collectively, these findings position the *Itga8-CreER* ^*T2*^ YAP/TAZ knockout as a robust and physiologically relevant model for studying vascular aneurysms and inflammation and support a critical role for YAP and TAZ in safeguarding the vasculature against disease.

Like other smooth muscles, bladder smooth muscle requires YAP/TAZ for contractile function. Using the *Itga8-CreER* ^*T2*^ driver, Liu L. et al. (2023) showed that YAP/TAZ deletion caused bladder enlargement and reduced contractile responses (Figure 2D). Transcript analysis revealed downregulation of contractile genes, muscarinic receptors, and transcriptional regulators (e.g., *Srf*, *Myocd*). Despite these changes, detrusor architecture remained intact with minimal remodeling, though perivascular inflammation was observed. YAP/TAZ therefore sustain transcriptional networks essential for SMC function across organs.

Together, these studies demonstrate that YAP and TAZ are indispensable for both the developmental establishment and adult maintenance of the SMC phenotype. YAP/TAZ depletion results in loss of contractile identity, functional impairment, and tissue degeneration in gastrointestinal, urogenital, and vascular systems. These phenotypes underscore the critical role of YAP and TAZ in smooth muscle homeostasis and suggest that maintaining SMC YAP/TAZ activity may protect against disease. Altogether, these findings also suggest that upstream Hippo signaling is critical in SMCs, but because mechanical inputs may control YAP and TAZ independently of Hippo, an important functional role cannot be taken for granted, and the relative roles of the Hippo kinases and adaptor proteins remains to be established using transgenic approaches.

Based on the account above, we predict that the partial inhibition of Hippo in SMCs, causing YAP/TAZ activation, would broadly target numerous SMC organs to favor contractility and increased contractile differentiation. In view of the centrality of Hippo in growth control, SMC-targeted Hippo inhibition may also promote growth of SMCs and remodeling of arteries (Kudryashova et al., 2016; Wang et al., 2012; Osman et al., 2019; Kimura et al., 2020). Based on data in other cell types (Sivaraj et al., 2024), a profibrotic response would not be surprising. Importantly, if selective inhibition of Hippo in SMCs was achievable in a clinical setting, this is predicted to counteract both aneurysms and possibly also plaque rupture, disease processes where biomechanics play a central role. If this occurs at the expense of increased scaring is not known. These and other hypotheses are testable using stringent, vascular SMC-specific, genetic inactivation of Hippo components, such as the MST or LATS kinases, and this has yet to be accomplished.

### 2.2 Target genes of YAP and TAZ in SMCs

YAP and TAZ are transcriptional co-regulators that primarily exert their biological functions through interaction with TEAD family transcription factors inside the nucleus. In contrast to the MYOCD/SRF complex, which activates a relatively fixed set of contractile genes regardless of cell type and typically acts at proximal promoters, YAP/TAZ/TEAD targets are more cell type-dependent and predominantly regulated *via* distal enhancers and alterations in chromatin accessibility (Monroe et al., 2019). This tissue specificity is shaped by factors that include cell-specific epigenetic landscapes, determining chromatin accessibility (Stein et al., 2015), and the presence or absence of specific transcriptional co-factors such as SMADs, AP-1, and MRTFs (Varelas et al., 2010; Zanconato et al., 2015; Kim T. et al., 2017). These diverse local signaling environments modulate the recruitment of YAP/TAZ to genomic loci, thus defining distinct transcriptional programs tailored to individual tissue contexts (Lopez-Hernandez et al., 2021).

**FIGURE 3:**
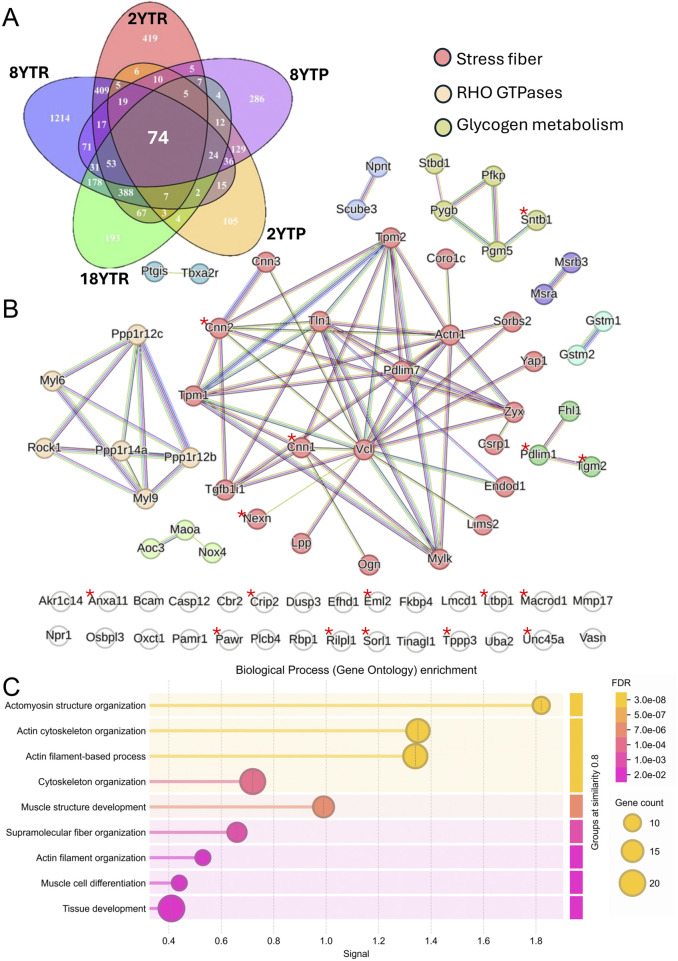
Target genes of YAP and TAZ in SMCs. The Venn diagram in **(A)** illustrates overlap between five datasets: RNA-seq from inducible vascular SMC-specific YAP/TAZ knockouts (KO) at 2 weeks (2YTR) and at 8 weeks (8YTR) after the first tamoxifen injection; proteomics from vascular SMC-specific YAP/TAZ KO at 2 weeks (2YTP) and 8 weeks (8YTP); and RNA-seq from SMC-specific YAP/TAZ KO 18 days after the first tamoxifen injection (18YTR). **(B)** shows protein–protein interaction network of 74 genes shared across the datasets. Markov clustering was performed in the STRING database with an inflation parameter of 3. Genes associated with stress fibers are highlighted in red, Rho GTPases in yellow, and glycogen metabolism in green. Genes marked with an asterisk show significant TEAD/YAP binding peaks (Q-value <1 × 10^−25^) within 5 kb of their transcription start site in both human and mouse ChIP-seq datasets obtained from ChIP-Atlas. **(C)** shows gene ontology of biological process enrichment analysis of the 74 shared genes using STRING database. Circle size reflects gene count, and color indicates false discovery rate (FDR).

By combining significantly downregulated transcripts/proteins from five independent datasets - including both RNA-sequencing and proteomic analyses at multiple time points following SMC-specific YAP/TAZ deletion (Daoud et al., 2022a; Arevalo Martinez et al., 2023; Wang et al., 2020) - we have identified 74 consistently downregulated genes (Figures 3A,B). Gene ontology analysis focusing on biological processes revealed strong associations with fundamental SMC functions such as cytoskeletal organization, muscle architecture, and differentiation (Figure 3C). Within the “stress fiber” cluster (red circles in the STRING network in Figure 3B), several key cytoskeletal components are represented, including *Actn1*, *Tln1*, *Vcl*, *Nexn*, and *Zyx*, along with the widely studied SMC differentiation markers calponin and myosin light chain kinase (*Cnn1*, *Mylk*) and the tropomyosins (*Tpm1*, *Tpm2*). A distinct Rho GTPase module (yellow in Figure 3B) includes myosin regulatory proteins (*Myl6*, *Myl9*), a key mediator of Rho-dependent signaling (*Rock1*), and myosin phosphatase subunits *Ppp1r12b* and *Ppp1r12c*. These target genes collectively underscore the critical role of YAP/TAZ in orchestrating contractile function and structural adaptations in SMCs.

Our STRING analysis also identified clusters related to oxidative stress, including *Nox4*, *Gstm1/Gstm2*, and *Maoa* (bottom left, bright green, Figure 3B), as well as a small set of genes relevant to glycogen metabolism (top right, dull green, Figure 3B), suggesting broader roles for YAP/TAZ in metabolic and redox homeostasis in SMCs. Notably, genes marked with an asterisk in Figure 3B indicate those demonstrating significant binding peaks (Q-value <1 × 10^−25^) within 5 kb of TEAD/YAP motifs in ChIP-seq datasets from both human and mouse studies acquired through the ChIP-Atlas Peak Browser. Furthermore, motif analysis of the upstream regions (−100 kb to −20 kb) of these 74 genes revealed significant enrichment for TEAD motifs using the JASPAR database, supporting the enhancer-mediated regulatory role of YAP/TAZ (Stein et al., 2015).

Loss of YAP/TAZ activity significantly impacts vascular pathophysiology, causing aneurysm formation (Daoud et al., 2022a; Arevalo Martinez et al., 2023; Sladitschek-Martens et al., 2022). If we were to prioritize YAP/TAZ target genes important for aneurysm protection, we would include *Myocd*, *Ctgf* (*Ccn2*), and *Lox*. *Myocd*, *Ccn2* and *Lox* are not represented among the 74 in Figure 3A, as they were not captured by mass-spectrometry (Arevalo Martinez et al., 2023), but they have been identified as target genes in independent work (Zhang et al., 2023; Creemers et al., 2006; Zhao et al., 2008), and knockout studies have demonstrated roles in aneurysm protection (Maki et al., 2002; Wang et al., 2023; Huang et al., 2015). Moreover, mutations or diminished expression of contractile-unit genes, such as *MYLK*, represented among the 74, are well-established causes of autosomal-dominant thoracic aortic aneurysms with dissection (Wallace et al., 2019; Hannuksela et al., 2016). Similarly, *TLN1* mutations were identified as a cause of aneurysms and dissections (Turley et al., 2019; Li et al., 2021), and *Mmp17* knockout predisposes mice to aortic aneurysms (Martín-Alonso et al., 2015). Other target genes may also contribute, including genes involved in extracellular matrix stabilization and cross-linking, such as *Tgm2*. Indeed, *Tgm2* expression is elevated in human abdominal aortic aneurysms and limits aortic dilation in mouse models through fibronectin cross-linking and matrix metalloproteinase inhibition (Shin et al., 2013; Griffin et al., 2021). Similarly, *ZYX* was associated with sporadic thoracic aneurysms, and its deficiency reduces SMC contractility, thus influencing vascular remodeling (Li et al., 2021).

Collectively, these findings illustrate a complex YAP/TAZ-dependent gene network that supports vascular integrity, mechanotransduction, and SMC function. While many of the identified genes likely contribute to aneurysm protection, the sheer number and diversity of targets complicate prioritization for therapeutic intervention. On the other hand, enhancing YAP/TAZ function in SMCs would represent a straightforward strategy for preventing aneurysm development and progression. However, the current data derive largely from studies in mouse SMCs, and further work is needed to definitively map YAP/TAZ targets in human vascular smooth muscle.

### 2.3 YAP and TAZ in endothelial cells

Despite the apparently lower expression of YAP and TAZ in undisturbed ECs compared to SMCs (Figure 1B), studies using EC-specific deletion and overexpression strategies have uncovered numerous critical roles for these transcriptional co-activators in vascular development and disease (Kobayashi et al., 2023). In 2015, Choi et al. were the first to demonstrate that Hippo signaling and YAP play essential roles in regulating angiogenesis (Choi et al., 2015). Subsequent work using floxed YAP/TAZ and LATS1/2 mice crossed with VE-cadherin-Cre (*Cdh5-CreER* ^*T2*^) mice revealed that inducible deletion of YAP and TAZ in adult ECs impairs vessel growth. Conversely, deletion of LATS1/2, leading to constitutive YAP/TAZ activation, caused hyperplastic vascular expansion (Kim J. et al., 2017).

Located at the blood-tissue interface, ECs use Hippo signaling to respond dynamically to hemodynamic forces. Dysregulation of EC function in the arterial wall is a known risk factor for atherosclerosis. It has been demonstrated that disturbed (turbulent) flow predisposes vessels to atherosclerotic plaque formation. Wang et al. reported increased YAP/TAZ activity in ECs exposed to disturbed flow, driving endothelial proliferation and inflammation (Wang K. C. et al., 2016). *In vivo* knockdown of YAP/TAZ using morpholino oligonucleotides reduced the inflammatory response and lessened atherogenesis. Further evidence using EC-specific CRISPR/Cas9-mediated knockdown of YAP, driven by the *Icam2* promoter and delivered *via* adeno-associated virus, showed that partial carotid ligation resulted in significantly reduced plaque formation (Wang K. C. et al., 2016). Notably, under conditions of laminar (unidirectional) flow, YAP/TAZ are phosphorylated and inactivated, even in the absence of LATS kinases, highlighting flow-dependent regulation (Wang K. C. et al., 2016). Still, multiple studies indicate that LATS remains critical for EC responsiveness to laminar shear stress (Xu et al., 2016; Cowdin et al., 2025). Additionally, YAP/TAZ inactivation can occur *via* integrin-Gα13 signaling, which activates MST1-LATS2 and suppresses atherogenesis (Wang L. et al., 2016). Conversely, EC-specific YAP/TAZ overexpression driven by the *Tie2-Cre* promoter in ApoE-deficient mice increased atherosclerotic burden (Wang L. et al., 2016).

Beyond genetic models, small-molecule inhibitors targeting the YAP/TAZ–TEAD interaction are emerging pharmacological tools, mainly in oncology (Baroja et al., 2024). Verteporfin is one such agent (Liu-Chittenden et al., 2012). When administered to ApoE-deficient Hutchinson-Gilford progeria syndrome mice on a high-fat diet, verteporfin reduced endothelial activation and atherosclerotic burden by inactivating YAP/TAZ (Barettino et al., 2024). However, given the diverse roles of Hippo signaling across cell types, systemic YAP/TAZ inhibition poses a significant risk of off-target effects (Baroja et al., 2024), highlighting a need for cell-specific targeting approaches.

One key insight from studies showing that YAP/TAZ activation in ECs promotes atherogenesis is that this effect depends on hypercholesterolemia. In transgenic models, hypercholesterolemia is generally required to observe pro-atherogenic effects (Glass and Witztum, 2001), suggesting that in its absence, YAP/TAZ activity in other vascular cell types, such as SMCs, may be accessible as a therapeutic target. Supporting this, EC-specific deletion of *Lats2* in normocholesterolemic mice (performed prior to growth plate closure) induces isolated myelofibrosis, primarily through SRF-driven endothelial-to-mesenchymal transition (Sivaraj et al., 2024), rather than promoting atherogenesis. Thus, in individuals with normal plasma cholesterol and stable, unstressed endothelial cells, endothelial-dependent pathology may be minimal, potentially allowing for selective, localized targeting of the Hippo pathway using LATS inhibitors.

An important point is that ECs and SMCs function interdependently to maintain arterial homeostasis. Communication occurs through soluble mediators such as nitric oxide (Furchgott and Zawadzki, 1980; Palmer et al., 1987) and endothelins (Yanagisawa et al., 1988), direct contact-dependent pathways, including NOTCH signaling (Hoglund and Majesky, 2012), and electrical or metabolic coupling *via* gap junctions (Sandow and Hill, 2000) and exosomes (Hergenreider et al., 2012). Consequently, genetic interventions targeting one cell type can influence the other, with potential systemic effects. How such crosstalk is altered in cell-specific knockout models remains to be fully defined.

### 2.4 The Hippo kinase LATS2 is likely SMC-enriched

Our work and that of others suggests SMC-specific inhibition of Hippo as a strategy for aneurysm protection (Daoud et al., 2022a; Arevalo Martinez et al., 2023; Sladitschek-Martens et al., 2022; Rippe et al., 2025). Inhibitors of both the MST and LATS kinases have been developed and can, in theory, be leveraged for this purpose. In SMCs, inhibition of Hippo is predicted to increase aneurysm-protective gene expression, including *Myocd*, *Ccn2* (*Ctgf*), and *Lox*, *via* YAP/TAZ (Daoud et al., 2022a; Rippe et al., 2025; Daoud et al., 2022b), but it seems improbable that this can be achieved with acceptable side effects considering the many functions in other cell types. An opportunity to bypass effects in other cell types would be to target an SMC-enriched Hippo constituent.

**FIGURE 4:**
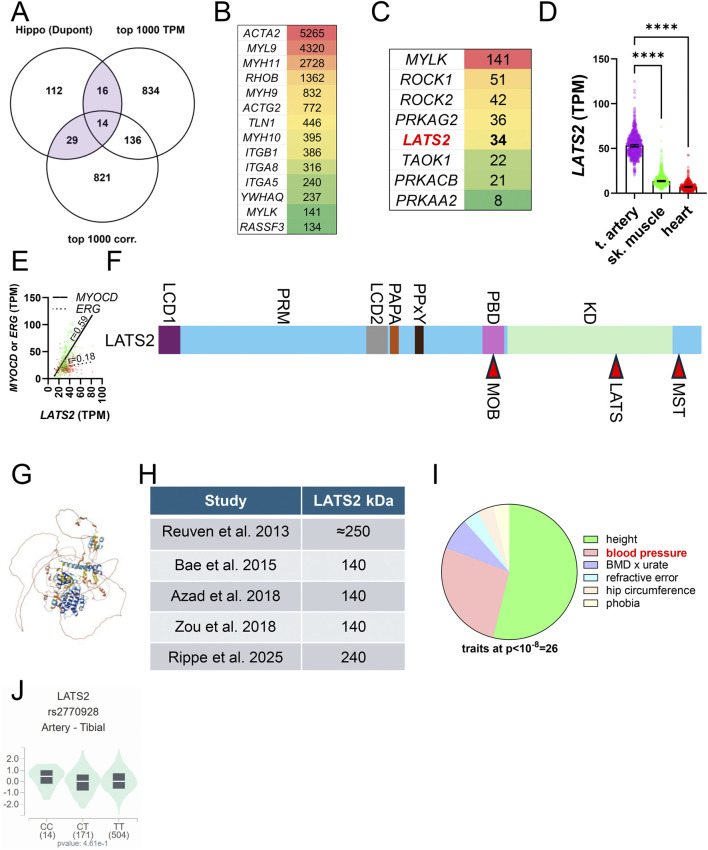
The core Hippo kinase LATS2 is likely SMC enriched. For initial insight into how Hippo is tailored to meet SMC-specific needs, we overlapped a comprehensive list of Hippo constituents with the top 1,000 transcripts in human coronary artery (GTEx data, TPM: transcripts per million) and with the 1,000 transcripts that correlate best with myocardin (MYOCD) across human tissues **(A)**. **(B)** shows the transcripts in the overlay of three datasets sorted on level of expression in transcripts per million (TPM). **(C)** shows kinases from the lower left overlap (Hippo and top 1,000 correlating). **(D)** shows that *LATS2* expression in tibial artery *versus* skeletal muscle and heart, and **(E)** shows that the SMC transcription factor *MYOCD* correlates better with *LATS2* than the EC transcription factor *ERG* does (coronary artery, GTEx data, n = 240). The overall domain organization of LATS2 is shown in **(F)**. LCD: low complexity domain, PRM: proline-rich motif, PAPA: proline-alanine repeats, PPxY: YAP and TAZ binding domain, PBD: protein binding domain that binds MOB1, KD: kinase domain. MST-dependent phosphorylation of LATS2 is on T1041 (red arrowhead in **(F)** leading to MOB1 binding. Autophosphorylation occurs on S835. **(G)** shows the Alphafold structure of LATS2, underscoring that long stretches of the protein are unstructured. The table in **(H)** highlights that LATS2 migrates at different molecular weights in different studies. Whether this depends on post-translational modifications or formation of an SDS-resistant complexes is not known. **(I)** shows pie-chart of traits associated with LATS2 variants (at p < 10^−8^) in the GWAS catalogue (https://www.ebi.ac.uk/gwas/genes/LATS2). Height is the most common trait, with blood pressure as a strong second. **(J)** shows a lack of significant association of rs2770928-T, coupled to blood pressure (p = 3 × 10^−10^) and height (8 × 10^−16^), with *LATS2* expression in arteries.

To approach SMC-enrichment, we overlapped a comprehensive list of Hippo constituents (Pocaterra et al., 2020) with the 1,000 transcripts that are most highly expressed in the human coronary artery (GTEx Consortium et al., 2013; GTEx Consortium, 2015), and with the 1,000 transcripts that correlate best with *MYOCD* across human SMC tissues, using the latter as a proxy of SMC-enrichment (Figure 4A). In the overlap of the three datasets, 14 transcripts were represented, with smooth muscle α-actin (*ACTA2*) having the highest level of expression (in transcripts per million, Figure 4B). It remains to be demonstrated that this actin isoform can control Hippo, but this seems likely given its critical role in acto-myosin driven contractility in arteries (Schildmeyer et al., 2000).

Because some SMC-enriched genes are expressed at low levels, transcripts in the overlap between Hippo and *MYOCD*-correlating transcripts are equally interesting (Figure 4A, bottom left overlap with 29 transcripts). This overlap features several kinases, including myosin light chain kinase (*MYLK*), the Rho-associated kinases (*ROCK1* and *ROCK2*), subunits of the AMP-activated kinase, as well as *LATS2* and *TAOK1* (Figure 4C). *LATS2* stands out because it is among the core Hippo kinases. The original *LATS2* cloning reports showed *LATS2* to be expressed at a high level in heart and skeletal muscle with an additional smaller isoform expressed in testis (Yabuta et al., 2000; Hori et al., 2000). With the completion of large-scale RNA-sequencing efforts using human tissues (GTEx Consortium et al., 2013; GTEx Consortium, 2015) it is now clear that *LATS2* expression is almost four-fold higher in arteries than it is in skeletal muscle and heart (Figure 4D). SMCs likely contribute more to *LATS2* expression in the arterial wall than ECs do, because the LATS2 protein expression increases dramatically on overexpression of myocardin (*MYOCD*) (Rippe et al., 2025), the transcription factor that governs SMC identity. Moreover, *LATS2* correlates better with *MYOCD* than with the EC transcription factor *ERG*, as exemplified in the human coronary artery in Figure 4E. We also note that the *LATS2* to *LATS1* expression ratio is extreme in arteries, reaching almost 4:1 as compared to 1:1 in liver. Altogether, these analyses and our recent finding that LATS2 is controlled by MYOCD/SRF (Rippe et al., 2025) suggest that Hippo in SMCs may depend disproportionally on LATS2. This, and other, yet to be defined, features of Hippo in SMCs, are predicted to be tailored to meet the demands of the biomechanically challenging environment of the arterial wall, exposed to the pressure generated by the heart throughout life.

Available LATS inhibitors target both LATS1 and LATS2, and given the high conservation of the kinase domain [85% amino acid identity, (Hori et al., 2000)], specificity is probably difficult to achieve through this part of the protein (labeled KD in Figure 4F). This inference is empirically supported by ongoing drug development efforts (Namoto et al., 2025). LATS2 further comprises two low complexity domains, with the first (LCD1) residing at the N-terminus (Figure 4F). The proline rich domain (PRM) following thereafter was recently shown to be involved in LATS2 condensate formation. The Alphafold structure predicts that a considerable part of the protein is unstructured (Figure 4G). The PPxY motif is important for binding to WW domains in YAP and TAZ. At the other end of the molecule is a protein binding domain important for binding to MOB1. Phosphorylation sites for MST and LATS kinases are located towards the C-terminal end (Figure 4F). The PAPA repeat consists of copies of the dipeptide proline-alanine and assists protein-protein interactions.

A thorough comparison of the domain organization of the two LATS homologues was provided elsewhere (Furth and Aylon, 2017). Among the important differences are that LATS2 contains a Nuclear Localization Signal that allows it to enter the nucleus more efficiently than LATS1, and that it features motifs that interact with p53 and nucleolar proteins, linking it to stress responses and genome integrity. In view of the apparently greater regulation of LATS2 than LATS1 by ubiquitination, it may be possible to selectively target LATS2 using Proteolysis Targeting Chimeras (PROTACs), but this remains to be demonstrated.

Early work indicated that Myc-tagged and overexpressed LATS2 migrates at an apparent molecular weight of 250 kDa in western blotting (Reuven et al., 2013). This has been challenged in subsequent studies demonstrating a 140 kDa isoform (Bae et al., 2015; Azad et al., 2018; Zou et al., 2018) using both cultured cells and cells acutely isolated from mice (Figure 4H). Our work showed that LATS2 in arteries (isolated acutely) migrates at 240 kDa (Rippe et al., 2025). Possible reasons for the weight discrepancy include complex formation (discussed below), but post-translational modifications may also be involved. LATS2 is known to be modified in numerous ways, including by Neddylation (Zou et al., 2018), ubiquitination (Bae et al., 2015; Ma et al., 2015), and OGlcNAcylation (Kim et al., 2020). Such factors could play a role in discrepant migration patterns in different studies and may allow for LATS2-specific pharmacological targeting. Altogether, these findings motivate further studies to explore and define the role of LATS2 in arterial SMCs using inducible and SMC-specific Cre deletion strategies.

### 2.5 Does human genetics support LATS2-targeting for vascular therapy?

An important yet unmet clinical need is medical therapies that limit or prevent the progressive expansion and rupture of aneurysms (Golledge, 2019). Based on the spontaneous, and breathtakingly fast, aneurysm development that occurs following genetic inactivation of YAP and TAZ in vascular SMCs (Zhang et al., 2023; Daoud et al., 2022a; Arevalo Martinez et al., 2023; Sladitschek-Martens et al., 2022), inhibition of Hippo in SMCs represents a possible avenue for artery protection. Such therapy, applied either locally, using stents, balloons or viral vectors, or systemically, by targeting SMC-unique aspects of Hippo, may not only halt aneurysm progression but could also be useful for preventing atherosclerotic plaque rupture. Small molecules that target Hippo are being developed at a steady pace and could be useful in this regard, but only if intended effects and side-effects exhibit distinct dose-dependencies (Namoto et al., 2024).

It was recently estimated that success for drugs that target specific biological mechanisms is roughly three-fold greater when there is genetic support in the human population (Minikel et al., 2024). We therefore examined genetic evidence connecting *LATS2* with human traits using the GWAS catalogue (https://www.ebi.ac.uk/gwas/genes/LATS2). There are 30 traits associated with *LATS2* variants (at p ≤ 10^−8^), with height featuring the largest number of associations (Figure 4I, green in the pie chart). This agrees with the growth-related phenotypes of *Hpo* mutants in *Drosophila*. The trait with the second most *LATS2* associations is blood pressure, including both systolic blood pressure and pulse pressure (Figure 4I, pink). This is consistent with our work in mice, showing reduction of blood pressure following YAP/TAZ knockout in SMCs (Arevalo Martinez et al., 2023). No associations with either aneurysms or coronary artery disease are represented in the GWAS catalogue at this significance threshold, but it should be noted that aortic aneurysms are comparatively rare, and the absence of an association may be due to lack of statistical power. None of the blood pressure-associated *LATS2* variants have a significant impact on arterial *LATS2* expression in GTEx, as exemplified by rs2770929 in tibial artery in Figure 4J. It therefore remains uncertain whether these associations are mediated by *LATS2* in the vascular wall. On a more positive note, however, there are no *LATS2* variants associated with cancer-related traits in the GWAS catalogue. At the very least, this opens for local LATS2 inhibition as a vascular therapy.

Variants at loci of other Hippo constituents are also represented by cardiovascular traits such as coronary artery disease in dyslipidemia (rs141435206-G at *STK3*/MST2, p = 4 × 10^−7^), migraine (rs12226331-T, *YAP1*, p = 2 × 10^−13^), and pulse pressure (rs12807220-A, *YAP1*, P = 8 × 10^−19^). Thus, taken together, these observations do not discourage LATS2-targeting in aneurysmal disease and they certainly motivate further work on the role of Hippo in SMC biology.

### 2.6 LATS2, ageing, and senescence

Age is a leading risk factor for cardiovascular disease (D'Agostino et al., 2008; Gonzalez et al., 2023; Liberale et al., 2025), with cellular senescence identified as a causal driver (Ungvari et al., 2018). Emerging evidence further shows that biological aging, molecular changes occurring across vascular cell types (Liberale et al., 2025), can diverge from chronological age, producing broad cardiovascular consequences. Nearly all cardiovascular diseases are influenced by aging, and aortic aneurysms are no exception, with risk rising 4.5-fold per additional decade of life (Pham et al., 2024). Consistent with aneurysm epidemiology, two independent studies reported reduced YAP/TAZ activity in the aging arterial wall (Rippe et al., 2025; Sladitschek-Martens et al., 2022). This raises the possibility that Hippo may undergo age-dependent remodeling, but this has yet to be demonstrated experimentally.

**FIGURE 5:**
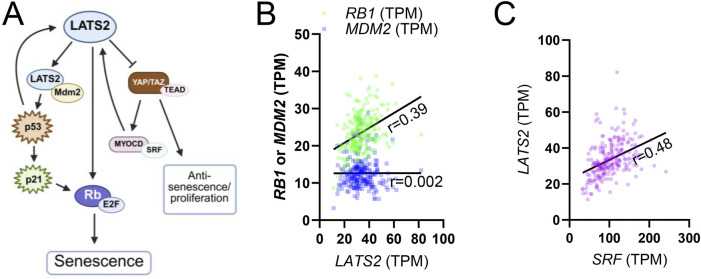
Role of LATS2 in cellular senescence. **(A)** shows a summary of how LATS2 affects cellular senescence through MDM2/P53 and Rb proteins. This summary is based on studies discussed in the main text. **(B)** shows that *LATS2* correlates with *RB1* in the human coronary artery (green symbols, p < 0.0001, n = 240), while no correlation is seen for *MDM2* (blue symbols). **(C)** shows correlation between *SRF* and *LATS2* for comparison (purple, p < 0.0001, n = 240).

As its name implies, “LArge Tumor Suppressor kinase 2” (LATS2) has emerged as a critical regulator of cell proliferation and tissue homeostasis. Through a role in promoting cellular senescence, a stable form of cell cycle arrest triggered by telomere attrition, DNA damage, or oncogenic stress, LATS2 may contribute to organismal aging. LATS2 is not among the panarterial aging transcripts identified in recent work (Rippe et al., 2025), but it may nonetheless promote senescence in an artery-specific manner *via* multiple mechanisms. One key pathway involves the stabilization of p53, a tumor suppressor that induces cell cycle arrest in response to DNA damage and oncogenic stress. LATS2 binds to MDM2, an E3 ubiquitin ligase that targets p53 for proteasomal degradation, thereby preventing p53 turnover and enabling activation of its downstream effector, p21 (Figure 5A) (Gao et al., 2025; Tschop et al., 2011). This activation in turn represses E2F target genes and initiates a sustained cell cycle arrest. Downstream of p53, the Retinoblastoma (Rb)/E2F pathway functions as a critical integrator of proliferative and checkpoint signals. Retinoblastoma (Rb) protein orchestrates stress-induced responses by modulating transcriptional programs involved in DNA repair, cell cycle blockade, and acquisition of senescence-associated phenotypes. As a master regulator of E2F activity, Rb determines whether cells proceed through the cell cycle or enter senescence (Gao et al., 2025). A kinase screen aimed at identifying regulators of Rb-induced senescence revealed LATS2 as a critical component, demonstrating that its activity is required for the establishment of the senescent phenotype (Tschop et al., 2011). Indeed, GTEx RNAseq data supports an association between *LATS2* and *RB1* (Rb) in human arteries (Figure 5B, coronary artery), albeit not as strong as that between *SRF* and *LATS2* (Figure 5C, coronary artery).

Global deletion of Lats2 in mice results in embryonic lethality around embryonic day 12.5, with phenotypes including atrial hyperplasia, consistent with its effect on growth especially in tissues of mesodermal origin. Also, mouse embryonic fibroblasts (MEFs) derived from Lats2-deficient embryos, bypass normal replicative senescence when cultured *in vitro* (McPherson et al., 2004). Interestingly, proliferative arrest, not overgrowth, appears to underlie embryonic death, underscoring the context-specific effects of Lats2. These data suggest that Lats2 can function either as a brake on proliferation or as a survival modulator depending on cell type, developmental stage, and extracellular cues. Adding to this complexity, LATS2 is paradoxically upregulated in certain cancers, where its expression correlates with increased metastatic potential, highlighting a dual role in tumor biology that may also extend to vascular pathophysiology (Furth and Aylon, 2017).

YAP/TAZ activity has been found to decline with ageing, suggesting an increasing impact of LATS2 with age (Sladitschek-Martens et al., 2022; Rippe et al., 2025). In our recent work, we found that expression of YAP/TAZ as well as the MYOCD/SRF transcriptional axis, both upstream regulators of SMC contractile phenotype, declines with age in human arteries (Rippe et al., 2025). Notably, overexpression of MYOCD in cultured vascular SMCs led to a significant increase in LATS2 expression (represented by an arrow in Figure 5A), suggesting that LATS2 lies downstream of this differentiation program and may be affected during vascular aging. Supporting this, knockout of SRF, used experimentally to mimic age-associated transcriptional decline, reduced LATS2 expression and provided protection against aneurysm formation *in vivo*. RNA-seq data (Rippe et al., 2025) show a modest negative correlation between LATS2 and age for the tibial artery, while showing an equally modest positive correlation in the aorta. This may be due to different transcriptional inputs in different arteries.

Taken together, these data suggest that while LATS2 expression itself does not undergo major changes with age, the age-related decline in regulators such as *SRF* may act to relieve proliferative restraint in vascular SMCs. Interestingly, this occurs in parallel with a decline in YAP/TAZ, key mediators of mechanosensitive proliferation and survival. Although the concurrent reduction in both pro- and anti-proliferative signals may seem paradoxical, it likely reflects a broader shift in the regulatory landscape of aging SMCs. In this altered state, decreased LATS2 activity may permit enhanced responsiveness to residual YAP/TAZ signaling or other proliferative stimuli, facilitating a limited reparative response under biomechanical stress. Alternatively, the simultaneous reduction of both axis may compromise cellular plasticity, contributing to maladaptive vascular remodeling or diminished repair. Targeted, cell-specific inhibition of LATS2 in vascular SMCs, may thus offer a promising therapeutic strategy to attenuate senescence, restore proliferative capacity, and promote vascular repair. Future studies are needed to define the spatial and temporal dynamics of LATS2 regulation in human arteries and to evaluate its feasibility as a therapeutic target in age-associated vascular diseases.

### 2.7 LATS kinases and heavy metal toxicity

The LATS kinases are best known for their ability to inactivate YAP and TAZ, but some functions are independent of YAP and TAZ, and governed by non-canonical downstream effectors (Furth and Aylon, 2017). Surprisingly, one of these is to sensitize cells to heavy metal-induced cell death. Han et al. (2022) found that knockout of LATS1 and LATS2 promoted viability of HEK293 cells in the presence of concentrations of cadmium (CdCl_2_) and zinc (ZnCl_2_) that caused almost complete loss of viability in control cells. This effect was not reproduced by constitutively active YAP (YAP5SA) or affected by YAP/TAZ knockout, indicating that YAP and TAZ are dispensable for heavy metal protection. LATS knockout increased transcripts that are important for heavy metal detoxification, including several metallothioneins and heavy metal transporters, and this depended on the metal regulatory transcription factor 1 (MTF1). It was demonstrated that LATS1-phosphorylates and inhibits MTF1. Importantly, the MST inhibitor XMU-MP-1, protected mice from cadmium induced death.

Like many other diseases, cardiovascular diseases are influenced by heavy metal exposure (Jomova and Valko, 2011; Lamas et al., 2023). For example, Cd, is present in cigarette smoke and elevated in smokers, and it increases the risk of peripheral arterial disease in man (Navas-Acien et al., 2004), and atherosclerosis in experimental animals (Messner et al., 2009). Therefore, current LATS inhibitors, which inhibit both LATS1 and LATS2, if used locally with the aim of targeting SMCs to protect arteries from the harmful effects of blood pressure, may have the added benefit of protecting from heavy metal-induced toxicity.

### 2.8 Hippo and biomolecular condensates

A biomolecular condensate is defined as a membrane-less, dynamic compartment within cells formed through phase separation of biomolecules, primarily proteins and nucleic acids (Shin and Brangwynne, 2017). It was demonstrated that both nuclear (Lu et al., 2020; Shao et al., 2024; Hao et al., 2024; Zhu et al., 2025) and cytoplasmic (Liu Q. et al., 2021; Wang et al., 2022; Liu J. et al., 2023; Bonello et al., 2023; Guo et al., 2024) biomolecular condensates play roles in Hippo-YAP/TAZ signaling. These condensates control pathway activation such as seen with altered cytoskeletal tension (Guo et al., 2024). An exciting possibility therefore is that condensates control YAP/TAZ activity in the vascular wall *in situ*, and that condensate formation can be targeted for therapy. Presently, it remains unknown whether Hippo signaling or YAP/TAZ form condensates in the vascular wall *in situ*, and the potential role of such condensates in vascular physiology or disease has not yet been explored.

**FIGURE 6:**
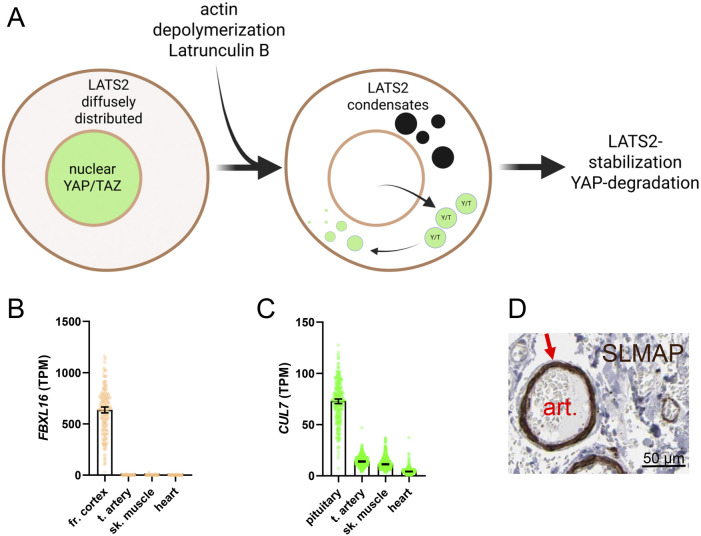
Biomolecular condensates and regulation of Hippo-YAP/TAZ. An emerging concept is that Hippo-YAP/TAZ activity is controlled by biomolecular condensates. Condensates arise by liquid-liquid phase separation. This increases the concentration of enzymes and substrates in confined volumes of the cell. One example is LATS2, found diffusely in the cytoplasm in control conditions **(A)**, cell to the left), with YAP and TAZ (green) being nuclear. On treatment with Latrunculin A, which depolymerizes actin, LATS2 redistributes to droplet-like condensates and YAP/TAZ leave nuclei. Condensate formation causes LATS2 stabilization and YAP degradation. The mechanism proposed for LATS2 stabilization involves protection from FBXL16-CUL7 mediated degradation. FBXL16 expression is essentially undetectable across muscle cell lineages **(B)**, data from GTEx) and CUL7 expression is low **(C)**, but this does not rule out LATS2 condensates forming through distinct mechanism. One possibility is through SLMAP, which is highly expressed in arteries **(D)**, from the Human Protein Atlas, HPA) and that forms Hippo-inactivating condensates. However, the major SLMAP isoform expressed in arteries probably lacks N-terminal sequences important condensate formation, and it remains to be demonstrated that the isoforms of SLMAP expressed in SMC can regulate Hippo.

Many Hippo constituents contain unstructured low-complexity domains (LCDs) that are important for condensate formation. LATS2 is no exception (LCD1 and LCD2, Figure 4F). A recent study found that depolymerization of actin filaments led to formation of LATS2 condensates that depended on the proline rich motif (PRM) (Qin et al., 2024). Condensate formation caused LATS2 stabilization and activation, causing YAP to degrade (Figure 6A). It was proposed that LATS2 condensates evade proteolysis by a CUL7-FBXL16 complex. This is a possible model for LATS2 regulation in the vascular wall, but there will surely be important differences. In fact, FBXL16 is essentially undetectable in arteries, and is primarily expressed in neural tissue (Figure 6B), while CUL7 expression is modest (Figure 6C). This argues that Hippo regulation is fine-tuned to serve tissue-specific needs, and that a lot remains to be learned about SMC-specific regulation.

One protein that forms Hippo-inactivating condensates is sarcolemmal membrane-associated protein (*SLMAP*) (Wang et al., 2022). SLMAP forms condensates that concentrate MSTs with the STRIPAK phosphatase complex, leading to MST dephosphorylation and inactivation. SLMAP is enriched in SMCs (Figure 6D shows SLMAP staining in a submucosal intestinal artery in brown, from the Human Protein Atlas), consistent with the reported regulation of this gene by myocardin (Sward et al., 2019; Liu et al., 2022). However, the SLMAP gene gives rise to several splice variants, with the major SMC isoform derived from late exons of the gene, and migrating at 40 kDa (Liu et al., 2022). Whether full length SLMAP is also regulated by myocardin remains to be determined. The SLMAP variant in SMCs therefore probably lacks N-terminal domains important for scaffolding functions. Consequently, high expression of SLMAP in SMCs cannot be equated with low constitutive Hippo activity. Indeed, as we have discussed above, overexpression of myocardin increases LATS2 expression and activates Hippo (Rippe et al., 2025). It remains an exciting possibility, however, that alternative splicing of SLMAP governs Hippo activity during SMC differentiation, and it is possible that unique aspects of condensate formation, involving either Hippo kinases, YAP/TAZ, or TEADs, may allow for SMC-specific Hippo targeting in disease.

### 2.9 The putative interactome of LATS2 in SMCs

**FIGURE 7:**
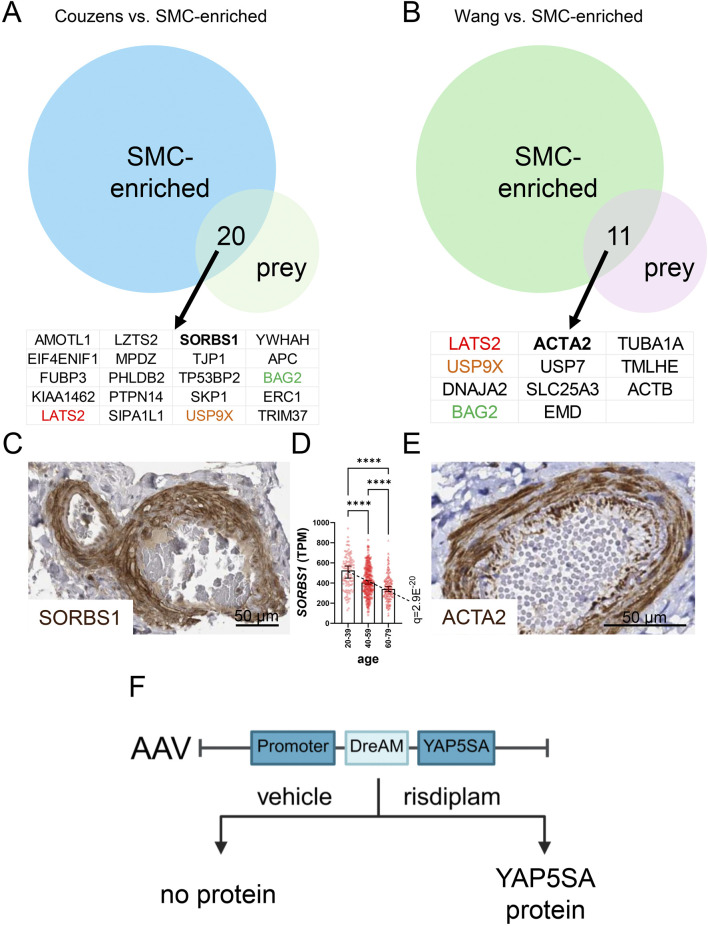
The LATS2 interactome reveals putative SMC-unique binding partners, and vector development for tunable YAP5SA expression may allow for SMC-specific targeting. Proteomic studies by Couzens et al. and by Wang et al. have mapped the interactome of LATS2. LATS2 and other Hippo constituents were used as bait to identify binding proteins (prey). When the LATS2 datasets are overlapped with transcripts that are enriched in SMCs, 20 and 11 gene products are represented in the overlaps **(A,B)**. Three were represented in both overlaps: LATS2, USP9X, and BAG2. SORBS1 and ACTA2, both of which are SMC-enriched proteins that are regulated by myocardin, are interesting candidates for SMC-specific regulation. **(C,E)** show positive staining (brown) for SORBS1 and ACTA2 in the media of human arteries (from the Human Protein Atlas). **(D)** shows the age-dependent decline of *SORBS1* expression in human tibial artery. Tunable YAP5SA expression in heart may overcome obstacles in heart repair. An AAV9 vector was therefore recently engineered **(F)**, that allows for brief YAP5SA protein expression in cardiomyocytes on short-term risdiplam administration.

Targeted studies have demonstrated binding between the LATS kinases and proteins beyond Hippo constituents, including the p53 regulator MDM2, and HSP90 (Furth and Aylon, 2017). More recent and unbiased proteomics studies have underscored that the interaction network is large and most likely cell type dependent (Wang W. et al., 2014; Couzens et al., 2013). To elucidate possible LATS2 binding partners relevant to mature SMCs, we overlapped SMC-enriched transcripts (transcripts in GTEx correlating with both *MYOCD* and *MYH11*) and LATS2 interactors identified by proteomics (Wang W. et al., 2014; Couzens et al., 2013) (Figures 7A,B). Among the proteins in the two overlaps, three were shared (colored gene symbols in Figures 7A,B). These included LATS2 itself, but also the ubiquitin-specific protease USP9X and the cochaperone BAG2. The latter proteins are potentially relevant for regulation of LATS2 stability in the arterial wall, with USP9X-LATS2 binding having been independently confirmed, and shown to control LATS2 deubiquitination and stability in other cells (Zhu C. et al., 2018). Although not common to both datasets, SORBS1 and ACTA2 emerge as possible regulators of LATS2 in SMCs. This is because both proteins are regulated by myocardin (Du et al., 2003; Liu et al., 2022; Yoshida et al., 2003), causing them to be highly expressed in vascular SMCs (Figures 7C–E show arterial staining in brown). Moreover, ACTA2 dynamics plays a role in SMC differentiation. Intriguingly, SORBS1 was reported to be critical for mechanotransduction in previous work (Kuroda et al., 2018; Holle et al., 2016) and our recent study (Rippe et al., 2025) identified SORBS1 as an age-dependent gene (Figure 7D). Unbiased proteomic experiments to identify LATS2 interactors in mature (or cultured and myocardin transduced) SMCs, under both healthy and diseased conditions, therefore emerge as a priority. This might confirm the LATS2-ACTA2 and LATS2-SORBS1 complexes but may also unravel novel SMC-specific Hippo targeting strategies that can be leveraged for vasoprotection.

## 3 Hippo-YAP/TAZ inhibitors and challenges for vasoprotective therapy

The idea that Hippo inhibition in SMCs may halt aneurysm progression, and perhaps even prevent atherosclerotic plaque rupture, is appealing, but there are obvious risks with this strategy that need to be overcome. This is illustrated by the pro-atherogenic shift expected from effects in ECs, at least in severe in dyslipidemia, but also by global *Lats1* knockout mice, reported to develop hyperplastic pituitary glands with endocrine disturbances and soft tissue sarcomas (St John et al., 1999). The latter findings alone argue forcefully against the untargeted and sustained inhibition of Hippo for any vascular therapy. Moreover, SMC-targeted YAP/TAZ activation may adversely affect gastrointestinal function (see above), even if this trade-off may be acceptable for any rapid and lethal disease.

Among the small molecules that can be exploited for modulating pathway activity are the older and less specific substances verteporfin, considered to inhibit the YAP-TEAD interaction (Liu-Chittenden et al., 2012), and GA-017 and TRULI, which are LATS inhibitors. Newer LATS inhibitors with higher specificity include TDI-011536 and NIBR-LTSi. TDI-011536 was reported to promote proliferation of cardiomyocytes after cryoinjury (Kastan et al., 2022), while NIBR-LTSi promoted stemness and proliferation in multiple organs (Namoto et al., 2024). Another compound that deserves mention is GNE-7883 (Hagenbeek et al., 2023), referred to as an allosteric pan-TEAD inhibitor, and that is evaluated for treatment of cancer, with a an initial focus on mesotheliomas where evidence for YAP/TAZ-driven malignancy is considerable. Thus, there are numerous molecules that can be considered for pathway targeting. NIBR-LTSi (Namoto et al., 2025), which is orally available, is an instructive example of the drawbacks of systemic LATS inhibition. NIBR-LTSi increased YAP activity, promoting stem cell expansion and blocking differentiation in mouse and human organoids. However, prolonged systemic administration caused adverse effects, including heart valve thickening and lung hyperplasia, limiting its therapeutic use (Namoto et al., 2024).

Another instructive example of limitations relates to the heart, where harnessing the ability of YAP/TAZ to promote cardiomyocyte proliferation and repair is considered a holy grail. In the heart, expression of constitutively active YAP (YAP5SA), which bypasses Hippo regulation, enhanced nuclear YAP activity and supported cardiomyocyte renewal. Yet, sustained expression triggered pathological reprogramming, leading to restrictive heart failure and death (Monroe et al., 2019), underscoring that length of treatment represents a critical concern. A drug-elicitable alternative splicing module (DreAM) was therefore developed for tunable expression of YAP5SA in heart (Chen et al., 2025). This adeno-associated viral (AAV9) vector operates on the principle that the splicing regulator risdiplam elicits a functional YAP5SA protein that is not generated in control conditions [Figure 7F, for exact vector design see (Chen et al., 2025)]. For heart expression, the *Tnnt2* promoter was used, and *in vivo* transduction plus short-term risdiplam administration dramatically improved survival after myocardial infarction, while bypassing heart failure and death seen in the long-term YAP-activation paradigm (Monroe et al., 2019; Chen et al., 2025). In theory, a vascular SMC-specific promoter (such as *Itga8*) could be used for transient YAP/TAZ activation in SMCs, but this vector remains to be constructed, and a meaningful effect must be demonstrated in relevant aneurysm models. Nonetheless, such vector development brings hope of achieving YAP/TAZ activation (or inhibition) in a cell-targetable and time-controlled manner for therapy of vascular disease.

## 4 Concluding remarks

Recent studies from many laboratories have underscored a life-sustaining role of YAP and TAZ in the arterial wall, particularly in SMCs. Here, YAP and TAZ protect from aneurysm formation, but what functional role the Hippo kinases play in SMCs currently represents a critical knowledge gap. Recent work has shown that YAP/TAZ-dependent artery protection abates with advancing age, a major non-modifiable risk factor for cardiovascular disease. In view of the considerable combined impact of hypertension and aging on the global burden of disease, it is of interest to hijack the inborn defense systems of the vasculature for vasoprotection. This may be achieved by targeting SMC-enriched Hippo constituents, possibly LATS2 or other pathway-regulatory mechanisms that are unique to this cell type. However, little is known about the organization and functional role of Hippo in SMCs. We therefore need to learn more about how Hippo is organized and regulated in arteries in general and in SMCs in particular. Work on many fronts, using cell-specific genetic approaches, imaging, proteomics, and vector design, is called for.
